# Renoprotective RAAS inhibition does not affect the association between worse renal function and higher plasma aldosterone levels

**DOI:** 10.1186/s12882-017-0789-x

**Published:** 2017-12-20

**Authors:** Christina M. Gant, Gozewijn D. Laverman, Liffert Vogt, Maartje C. J. Slagman, Hiddo J. L. Heerspink, Femke Waanders, Marc H. Hemmelder, Gerjan Navis, Holland Nephrology Study (HONEST) Network

**Affiliations:** 1 Department of Internal Medicine, Division of Nephrology, University of Groningen, University Medical Centre Groningen, Hanzeplein 1, 9713 GZ Groningen, The Netherlands; 2 Department of Internal Medicine/Nephrology, ZGT Hospital, Zilvermeeuw 1, 7609 PP Almelo, The Netherlands; 3 Department of Internal Medicine, Academic Medical Centre, University of Amsterdam, Meibergdreef 9, 1105 AZ Amsterdam, The Netherlands; 4 Department of Clinical Pharmacy and Pharmacology, University Medical Centre Groningen, University of Groningen, Hanzeplein 1, 9713 GZ Groningen, The Netherlands; 5 Department of Internal Medicine/Nephrology, Isala Hospital, Dokter van Heesweg 2, 8025 AB Zwolle, The Netherlands; 6 Department of Internal Medicine/Nephrology, Medical Centre Leeuwarden, Henri Dunantweg 2, 8934 AD Leeuwarden, The Netherlands

**Keywords:** Aldosterone, Chronic kidney disease, Creatinine clearance, RAAS inhibition, Systolic blood pressure, Dietary sodium restriction

## Abstract

**Background:**

Aldosterone is elevated in chronic kidney disease (CKD) and may be involved in hypertension. Surprisingly, the determinants of the plasma aldosterone concentration (PAC) and its role in hypertension are not well studied in CKD. Therefore, we studied the determinants of aldosterone and its association with blood pressure in CKD patients. We also studied this during renin-angiotensin-aldosterone system inhibition (RAASi) to establish clinical relevance, as RAASi is the treatment of choice in CKD with albuminuria.

**Methods:**

We performed a post-hoc analysis on data from a randomized controlled double blind cross-over trial in non-diabetic CKD patients (*n* = 33, creatinine clearance (CrCl) 85 (75–95) ml/min, proteinuria 3.2 (2.5–4.0) g/day). Patients were treated with losartan 100 mg (ARB), and ARB + hydrochlorothiazide 25 mg (HCT), during both a regular (200 ± 10 mmol Na^+^/day) and low (89 ± 8 mmol Na^+^/day) dietary sodium intake, in 6-week study periods. PAC data at the end of each study period were analyzed. The association between PAC and blood pressure was analyzed continuously, and according to PAC above or below the median.

**Results:**

Lower CrCl was correlated with higher PAC during placebo as well as during ARB (β = −1.213, *P* = 0.008 and β = −1.090, *P* = 0.010). Higher PAC was not explained by high renin, illustrated by a comparable association between CrCl and the aldosterone-to-renin ratio. The association between lower CrCl and higher PAC was also found in a second study with single RAASi with ACE inhibition (ACEi; lisinopril 40 mg/day), and dual RAASi (lisinopril 40 mg/day + valsartan 320 mg/day). Higher PAC was associated with a higher systolic blood pressure (P = 0.010) during different study periods. Only during maximal treatment with ARB + HCT + dietary sodium restriction, blood pressure was no longer different in subjects with a PAC above and below the median.

**Conclusions:**

In CKD patients with a standardized regular sodium intake, worse renal function is associated with a higher aldosterone, untreated and during RAASi with either ARB, ACEi, or both. Furthermore, higher aldosterone is associated with higher blood pressure, which can be treated with the combination of RAASi, HCT and dietary sodium restriction.

The first study was performed before it was standard to register trials and the study was not retrospectively registered. The second study was registered in the Netherlands Trial Register on the 5th of May 2006 (NTR675).

**Electronic supplementary material:**

The online version of this article (10.1186/s12882-017-0789-x) contains supplementary material, which is available to authorized users.

## Background

Aldosterone is involved in sodium and volume homeostasis, and consequently regulation of extracellular volume and blood pressure. It has a main role in the homeostatic response to volume depletion, where higher aldosterone levels contribute to renal retention of sodium and water, and followingly restoration of the extracellular volume. In chronic kidney disease (CKD), derangement of volume status and hypertension are common. Several studies indicate an inverse association between plasma aldosterone levels and creatinine clearance [1–4].

Inappropriately high aldosterone levels in CKD have been suggested to contribute to CKD-associated hypertension, as well as to progressive kidney damage by direct profibrotic effects of aldosterone [5–7]. Inhibition of the renin-angiotensin-aldosterone system is a cornerstone of therapy in CKD, for treatment of hypertension and proteinuria [8]. Whether RAAS inhibition (RAASi) ameliorates the high aldosterone levels associated with CKD, however, has not been systemically assessed.

Therefore, we aim to investigate, first, the determinants of the plasma aldosterone concentration (PAC) and aldosterone-to-renin ratio (ARR) in non-diabetic CKD, both without and with RAAS inhibition (RAASi). Second, we aim to investigate whether in CKD, high aldosterone is associated with blood pressure.

To this purpose, we studied the association between PAC, ARR and renal function during different treatment conditions in a previously performed randomized controlled trial. Here, ARB (losartan 100 mg/day) and placebo were compared in combination with dietary sodium restriction and HCT (hydrochlorothiazide 25 mg/day) in non-diabetic proteinuric CKD patients [9]. In this analysis we found that during ARB, renal function was negatively correlated with PAC, similarly as during placebo. To provide independent confirmation of the lack of efficacy of RAASi on the association between lower renal function and higher PAC, we analysed the association between PAC, ARR and creatinine clearance in a second study with comparable design. This second study had study periods of single RAASi with ACE inhibition (ACEi), and dual RAASi with ACEi + ARB, both during a standardized regular and low sodium intake [10].

## Methods

### Patients and study design

This is a post-hoc analysis of a previously performed randomized controlled cross-over trial, the protocol is described in detail elsewhere [9]. For all clinical experimentation, approval was obtained from the Institutional Review Board (Medical Ethics Committee of the University Medical Centre Groningen) with oversight authority for the protection of human research subjects. The study was performed according to the Declaration of Helsinki of 1975, as revised in 2000. All participants gave written informed consent before study-related procedures were performed. In short, patients had stable proteinuria due to non-diabetic CKD, were middle aged, and had stable creatinine clearance (>30 mL/min, <6 mL/min/year decline). Patients were randomized to a low sodium diet (target sodium intake 50 mmol Na^+^/day; approximately 1200 mg Na^+^/day or 3 g NaCl/day) or a regular sodium diet (target sodium intake 200 mmol Na^+^/day; 4800 mg Na+/day or 12 g NaCl/day). Dietary counselling was aimed at avoiding salty foods while keeping other dietary habits unchanged. Patients remained on the assigned diet for 18 weeks, consisting of three consecutive 6-week study periods. Placebo was always as the first treatment, followed by ARB (losartan 100 mg/day), and ARB + HCT (losartan 100 mg/day + hydrochlorothiazide 25 mg/day), where ARB and ARB + HCT were given in random order. After 18 weeks, the patients changed their diet and the three 6-week periods were repeated in the same order as the first three 6-week periods. Therefore, in total there were 4 possible treatment sequences. Additional antihypertensive drugs were allowed if required; these were kept constant during the study.

### Independent confirmation: No effect RAASi on the association of creatinine clearance and PAC

We assessed the effect of single RAASi with ACEi vs dual RAASi with ACEi + ARB, respectively, on the association between PAC, ARR and creatinine clearance in an independent second study, with a comparable cross-over design during different sodium intakes. The protocol is described in detail elsewhere [10]. Patients had comparable non-diabetic CKD with stable proteinuria and creatinine clearance (>30 mL/min, <6 mL/min/year decline) In this trial (*n* = 45) the 6-week study periods consisted of single RAASi (lisinopril 40 mg/day) and dual RAASi (lisinopril 40 mg/day + valsartan 320 mg/day), both during a regular sodium diet (target 200 mmol Na^+^/day) and a low sodium diet (target 50 mmol Na^+^/day), in random order. As 7 patients participated in both trials, we excluded them from the second analysis, to avoid duplication of the results.

### Measurements and calculations

Proteinuria was measured by the pyrogallol red-molybdate method in 24-h urine samples. Blood pressure was determined at 1 min intervals with an automatic device (Dinamap, GE Medical Systems, Milwaukee, WI, USA) with the patient in a supine position. After 15 min of measurements, we used the mean of the last three readings for further analyses. Dietary sodium intake was assessed from 24-h urinary sodium excretion. We calculated creatinine clearance from creatinine concentrations in plasma and in 24-h urine samples. Peripheral blood was drawn by venipuncture, and aliquots from serum were stored (−80 °C) until plasma aldosterone concentration (PAC) and active plasma renin concentration (APRC) analysis. Samples were taken in a standardized manner, with the patient in a supine position, and were directly put on ice. Aldosterone was measured with a commercially available RIA kit (Diagnostic Products Corp., Los Angeles, CA). Plasma renin activity was measured as described previously with a RIA that detects the amount of angiotensin I produced per hour in the presence of excess angiotensinogen (nanograms of angiotensin I produced per millilitre of plasma per hour) [11]. This assay measures the enzymatic activity of active plasma renin in the presence of an excess of its (exogenous) substrate.

In the second study, renin was determined using direct renin measurements, in this respect, data regarding the aldosterone-to-renin ratio in both studies could not be pooled. The remaining measurements and calculations in the second study used for the independent analysis were performed equally [10].

### Statistics

All 33 patients were included in our data analysis, and are presented here. Skewed variables were natural log (LN) transformed in order to achieve a normal distribution for linear regression and linear mixed model analyses. Data is given as mean with standard deviation when normally distributed, and geometric mean with 95% confidence interval when data is skewed. SPSS 22.0 for Windows was used for all analyses.

To assess the determinants of PAC and ARR, linear regression analyses were performed. The log transformed PAC and ARR were used as the dependent factors. Analysed variables are: age, gender, body mass index, serum sodium, 24-h urinary sodium excretion, serum potassium, 24-h urinary potassium excretion, log transformed plasma renin activity, log transformed proteinuria and log transformed creatinine clearance. This analysis was equally performed in data from the second study.

Treatment effects were determined using linear mixed model analyses, with Sidak post-hoc analyses to localize the differences. Statistical significance was assumed at the 5% level of probability. The covariance structure for all mixed model analyses was based on Akaike’s information criterion. The association between PAC, and systolic blood pressure (SBP) was studied using linear mixed model analyses. First the association between aldosterone and SBP, and possible confounders (gender, age, creatinine clearance), were tested univariately. In univariate analysis, SBP was the dependent variable, and sodium diet, ARB, HCT, their interaction (sodium diet x ARB x HCT), and log transformed PAC (or gender, age, creatinine clearance) were fixed factors. Subjects was used as a random factor. Covariates with a *P* < 0.100 were included in the multivariate mixed model.

To ensure there were no carryover effects from the different treatment regimens, we performed linear mixed model analyses. Log transformed PAC was the dependent factor, and treatment and sequence as well as their interaction (treatment x sequence) were fixed factors. We did similar analyses with log transformed ARR, and systolic blood pressure as the dependent factors. Carry-over effects were not detected (treatment x sequence was not a statistically significant predictor), and for the sake of conciseness these data are not shown.

## Results

### Baseline characteristics and responses to treatment

Patient characteristics and adherence to the program have been described previously [9], (see also Additional file 1: Table S1). In total 33 participants have completed the study. During regular dietary sodium intake, mean 24-h urinary sodium excretion was 197 ± 63 mmol Na^+^/day (approximately 4600 mg Na^+^/day or 12 g NaCl/day), and during low dietary sodium intake (LS) this was 92 ± 46 mmol Na^+^/day (approximately 2100 mg Na^+^/day or 5 g NaCl/day; Table 1). Systolic blood pressure dropped stepwise with the lowest value on ARB + HCT + LS, whereas diastolic blood pressure was lowest on ARB + HCT on either sodium intake. Creatinine clearance fell significantly after sodium restriction during both ARB and ARB + HCT treatment. Proteinuria declined after each additional treatment step, with the lowest value in ARB + HCT + LS.

**Table 1:** Clinical and biochemical parameters during different study periods

		Placebo	ARB	ARB + HCT
Systolic blood pressure (mmHg)	RS	143 (4)	135 (3)^†^	125 (3)^†‡^
	LS	136 (3)*	128 (3)*^†^	121 (2)^†‡^
Diastolic blood pressure (mmHg)	RS	86 (2)	80 (2)^†^	75 (1)^†‡^
	LS	83 (1)*	78 (1)^†^	74 (1)^†‡^
Plasma potassium concentration (mmol/l)	RS	4.3 ± 0.1	4.4 ± 0.1	4.0 ± 0.1^†‡^
	LS	4.3 ± 0.1	4.5 ± 0.1^†^	4.0 ± 0.1^†‡^
24 h sodium excretion (mmol/day)	RS	200 ± 10	197 ± 11	193 ± 11
	LS	89 ± 10*	92 ± 8*	93 ± 8*
Creatinine clearance (ml/min)	RS	85 (73–98)	90 (77–106)	81 (69–95)^‡^
	LS	80 (67–94)	79 (67–93)*	72 (61–85)*
Proteinuria (g/day)	RS	3.4 (2.6–4.3)	2.3 (1.8–3.0)^†^	1.3 (1.0–1.7)^†‡^
	LS	2.3 (1.7–3.1)*	1.3 (0.9–1.7)^†^*	0.9 (0.6–1.2)*^†‡^
Active plasma renin concentration (ng AI/ml x h)	RS	15 (12–18)	33 (23–45)^†^	64 (47–87)^†‡^
	LS	19 (15–23)	51 (38–70)*^†^	130 (103–163)*^†‡^
Plasma aldosterone concentration (ng/l)	RS	66 (48–91)	57 (41–79)	96 (74–125)^‡^
	LS	106 (73–152)	113 (86–149)*	164 (129–207)*^†‡^
Aldosterone-to-renin ratio (ng/ng AI × h)	RS	19 (13–26)	7 (4–11)^†^	6 (4–9)^†^
	LS	22 (14–32)	9 (6–13)^†^	5 (4–7)^†‡^

*ARB* angiotensin receptor blocker (losartan 100 mg/day), *HCT* hydrochlorothiazide (25 mg/day), *RS* regular sodium intake, *LS* low sodium intake

*P < 0.05 vs regular sodium on same treatment (effect of LS)

^†^ *P* < 0.05 vs placebo on same sodium diet

^‡^ *P* < 0.05 vs ARB on same sodium diet (effect of HCT)

### PAC and ARR during different treatment conditions

PAC did not change during ARB as compared to placebo, neither during the regular nor during low sodium intake (*P* = 0.9 and *P* > 0.999 respectively, Table 1). However, the ARR declined significantly during ARB in both sodium intakes, illustrating pharmacological inhibition of the RAAS by ARB. The addition of HCT to ARB increased PAC in both sodium intakes (*P* < 0.01 and *P* = 0.01), illustrating reduction of extracellular fluid volume. Dietary sodium restriction was associated with a higher PAC during ARB and ARB + HCT, but not during placebo treatment.

### Determinants of PAC and ARR, during placebo and single RAASi with ARB

**Fig. 1:**
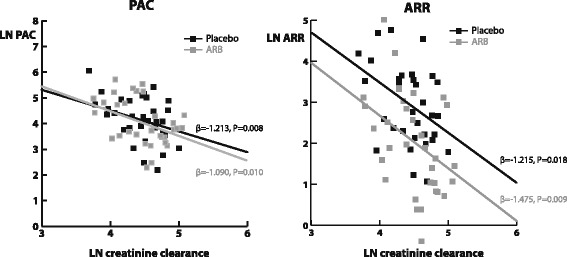
Correlation between creatinine clearance and the plasma aldosterone concentration (left panel), and aldosterone-to-renin ratio (right panel) during placebo and ARB treatment, during a regular sodium intake. Creatinine clearance is significantly and negatively correlated with PAC, and this correlation is similar during placebo and ARB treatment. ARR is similarly, and negatively correlated with creatinine clearance. During RAASi the regression line is parallel and shifted downwards. PAC: plasma aldosterone concentration; ARR: aldosterone-to-renin ratio; ARB: angiotensin receptor blocker (losartan 100 mg/day)

As anticipated, during placebo, creatinine clearance was negatively and significantly associated with PAC (β = −1.213, *P* = 0.008; Table 2). During ARB, the negative correlation between creatinine clearance and PAC was similarly present (Fig. 1). Neither plasma renin activity (placebo β = 0.274, *P* = 0.29; ARB β = 0.171, *P* = 0.30), nor serum potassium (placebo β = 0.346, *P* = 0.42; ARB β = 0.011, *P* = 0.9) were significant determinants of PAC in either treatment condition. During low dietary sodium intake results were virtually similar. In multivariate analysis, when adjusting for age and gender, creatinine clearance remained the only significant predictor of PAC in both treatment conditions (Additional file 2: Table S2).

**Table 2:** Determinants of the plasma aldosterone concentration and aldosterone-to-renin ratio during placebo and during ARB, during a regular sodium intake

	Plasma aldosterone concentration				Aldosterone-to-renin ratio			
	Placebo		ARB		Placebo		ARB	
	β	*P*-value	β	*P*-value	β	*P*-value	β	*P*-value
Age (years)	0.011	0.41	0.006	0.67	0.027	0.06	0.350	0.049
Gender (women)	−0.030	0.94	0.492	0.17	0.494	0.22	0.760	0.11
BMI baseline (kg/m^2^)	0.009	0.81	−0.009	0.80	0.015	0.70	0.029	0.56
Serum sodium (mmol/l)	0.041	0.53	−0.086	0.13	0.034	0.64	0.260	0.74
24 h urinary sodium excretion (mmol/day)	0.001	0.85	−0.002	0.35	<0.001	0.93	−0.003	0.38
Serum potassium (mmol/l)	0.346	0.42	0.011	0.98	0.377	0.42	0.355	0.61
24 h urinary potassium excretion (mmol/day)	−0.001	0.88	−0.003	0.60	<−0.001	>0.99	−0.004	0.64
LN Proteinuria (g/day)	0.078	0.75	−0.135	0.58	0.003	0.99	0.075	0.82
LN Creatinine clearance (ml/min)	−1.213	0.008	−1.090	0.01	−1.215	0.02	−1.475	0.009
LN Active plasma renin concentration (ng AI/ml x h)	0.274	0.29	0.171	0.30				

*ARB* angiotensin receptor blocker (losartan 100 mg/day), *LN* natural logarithm

Predictors of the aldosterone-to-renin ratio (ARR) were similar to those of PAC (Table 2, Fig. 1). Again, as anticipated, during placebo treatment, creatinine clearance was negatively significantly correlated with ARR (β = −1.215, *P* = 0.02). During ARB, the association was similarly present (β = −1.475, *P* = 0.01), although the regression line was shifted downwards, illustrating pharmacological inhibition of the RAAS by ARB treatment (Fig. 1). During low dietary sodium intake results were virtually similar.

### Second study. Association between CrCL and PAC during single RAASi with ACEi and dual RAASi

In a second study, we assessed the effect of single RAASi with ACEi (lisinopril 40 mg/day) and dual RAASi (lisinopril 40 mg/day + valsartan 320 mg/day) on the negative association between creatinine clearance and PAC and ARR during a standardized regular sodium intake [10]. Patient characteristics are shown in Additional file 1: Table S1. The plasma aldosterone concentration during single RAASi with lisinopril was 60 (73–89) ng/L, and did not differ from the PAC in the original study during single RAASi with losartan (*P* = 0.543). During dual RAASi the PAC did not change (71 (59–86) ng/L, *P* = 0.993). Also in this second study, creatinine clearance was significantly and negatively correlated with PAC during single RAASi, albeit with lisinopril (β = −0.646, *P* < 0.003). With ARR it did not quite reach statistical significance (β = −1.019, *P* = 0.07). During dual RAASi treatment with lisinopril and valsartan, creatinine clearance was significantly and negatively correlated with both PAC and ARR (β = −0.805, P = <0.001 and β = −2.020, *P* = 0.005 respectively).

### Aldosterone and blood pressure

To study the association between PAC and systolic blood pressure (SBP) in different treatment conditions, we performed linear mixed model analyses. Sodium diet ARB, HCT, gender and log transformed aldosterone were significant predictors of SBP (Table 3), demonstrating an association between PAC and SBP, which was independent of creatinine clearance, age, and gender.

**Table 3:** Linear mixed model analysis on the association between different predictors and the systolic blood pressure

Parameter	Parameter estimate	*P*-value
Low sodium diet	−7.753	<0.001
ARB	−8.385	<0.001
HCT	−10.537	0.008
Gender (women)	−7.705	0.01
Age (years)	0.159	0.16
LN Creatinine Clearance (ml/min)	−5.513	0.14
LN Aldosterone (ng/l)	6.477	<0.001

*ARB* losartan 100 mg/day, *HCT* hydrochlorothiazide 25 mg/day, *PAC* plasma aldosterone concentration

**Fig. 2:**
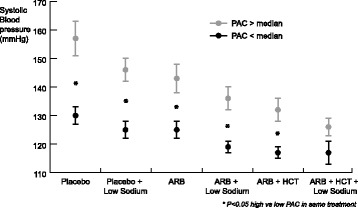
Systolic blood pressure during different treatment conditions in patients with a PAC above the median, and a PAC below the median. SBP is higher in the high PAC group in all treatment conditions, but the difference tends to decrease when treatment was intensified. Error bars represent standard error of the mean. SBP: systolic blood pressure; PAC: plasma aldosterone concentration; ARB: angiotensin receptor blocker (losartan 100 mg/day); LS: dietary sodium restriction; HCT: hydrochlorothiazide (25 mg/day)

To visualize the association between PAC and SBP, we dichotomized the group in subjects with a baseline PAC above the median and a baseline PAC below the median, also shown in Fig. 2. During placebo, SBP was significantly higher in the high PAC group than in the low PAC group (157 ± 25 mmHg vs 130 ± 13; *P* = 0.001). This difference was also seen during placebo + LS (146 ± 17 vs 126 ± 12; P = 0.001), ARB (143 ± 20 vs 125 ± 12; P = 0.005), during ARB + LS (136 ± 14 vs 119 ± 9; *P* < 0.001) and during ARB + HCT (132 ± 17 vs 117 ± 9; *P* = 0.004). Only during the most extensive treatment regimen of ARB + HCT + LS, there was no statistical significant SBP difference between the groups, while the mean blood pressure is still higher is patients with a high PAC (126 ± 13 vs 117 ± 14; *P* = 0.05).

## Discussion

In this study, we found that pharmacological effective RAASi did not affect the correlation between worse renal function and higher aldosterone. This was true for single RAASi with either ARB or ACEi, as well as for dual RAASi. This indicates that the association between creatinine clearance and aldosterone levels is not susceptible to RAASi, not even with dual inhibition. Furthermore, higher PAC was associated with a higher systolic blood pressure, independent of age, gender and creatinine clearance. During stepwise treatment with losartan, hydrochlorothiazide and dietary sodium restriction this difference decreased to the point where this difference was no longer statistically significant at the final treatment step (losartan, hydrochlorothiazide and a low sodium diet).

The association between lower creatinine clearance and higher PAC and ARR we observed is consistent with older data [1]. However, the older data were obtained before the era of RAASi, and did not control for sodium status. The authors reported that in patients with reduced creatinine clearance of more than 50% of normal, PAC was high while serum potassium and APRC were within normal values. Our data are consistent, and extend these findings by demonstrating that the association holds during RAASi with either ARB or ACEi. This is relevant to show, as RAASi is the treatment of choice for CKD with albuminuria. In line, Hannemann et al. describe an inverse association between renal function and aldosterone in a large general population cohort uncontrolled for sodium status [3]. McQuarrie et al. however, found that sodium status, and not renal function determined urinary mineralocorticoid excretion in CKD [12]. In our study sodium intake was standardized, which allowed to examine the effect of renal function and RAASi on PAC.

To our knowledge, the association we describe between high aldosterone and blood pressure has not been described before in CKD patients under RAASi. These data suggest that treatment with MRA is efficacious to treat hypertension in CKD. Indeed, a meta-analysis of patients with mild or moderate CKD has shown that intervention with a mineralocorticoid receptor antagonist (MRA) as adjunct to RAASi reduces blood pressure [13]. In resistant hypertensive patients without CKD, the association between high aldosterone and volume expansion has been previously established [14]. The recent PATHWAY study has shown that in treatment-resistant hypertension spironolactone as an adjunct treatment is more efficacious in lowering blood pressure than doxazosin or bisoprolol [15]. However, in this study the blood pressure lowering effect of MRA was correlated with baseline plasma renin, but not plasma aldosterone levels. In addition, in obesity, which is a different pathophysiological condition where aldosterone levels are inappropriately increased, aldosterone has also been shown to be associated with sodium-sensitive hypertension [16–19]. We show for the first time that also in CKD, high aldosterone levels are associated with higher blood pressure, even despite RAASi.

The mechanism underlying the consistent association between lower renal function and high PAC during RAASi is unknown. The parallel association between worse renal function and ARR, and the persistence of both associations during RAASi, with either ARB or ACEi, suggest that the higher aldosterone with lower renal function is not due to renin activation. This is further supported by the persistence during dual RAASi, which makes it unlikely that the association persists due insufficient pharmacological blockade of angiotensin II and the RAAS. Furthermore, the association between worse renal function and PAC was similarly present during ARB and ACEi, which differ in various pharmacological aspects; i.e. during ARB there is a compensatory rise in angiotensin II and increased angiotensin II type 2 receptor activation, and during ACEi bradykinin levels may increase. This renders it unlikely that pharmacologically different modes of RAASi play a role in the association between renal function and PAC. The consistent association between renal function and PAC during different modes of RAASi may suggest other mechanisms and pathways are involved, which are not susceptible to RAASi. Recent data show a link between aldosterone and the calcium-phosphate-PTH-FGF23 axis [20]. There is evidence of a correlation between increased phosphaturic hormone fibroblast growth factor 23 (FGF23) and increased aldosterone through unknown mechanisms [21, 22]. Also, a bidirectional interplay between PTH and aldosterone has been suggested [23–26]. Alternatively, increased intrarenal RAAS activation might be involved. In this context, it is interesting to note that it has been shown that a putative marker for intrarenal RAAS activation, is inversely correlated with renal function in diabetic kidney disease [27]. Additionally, the link between low creatinine clearance and high aldosterone could be a compensatory response to the reduced clearance of potassium that occurs when glomerular filtration rate falls, however, as the plasma potassium concentration is very tightly regulated, this hypothesis could not be tested in this study while data regarding potassium intake in respect to potassium excretion is unknown. Another possible explanation could be that it is the other way around, so that the high PAC leads to a decline in renal function due to the pro-fibrotic effects of aldosterone [5–7]. Several studies found a high PAC and ARR to be associated with the development of CKD [28, 29].

What are the implications of our study? Our data show that patients with renal function impairment, over a range from mild to moderately impaired, have high aldosterone, which is associated with higher blood pressure, even despite RAASi. Thus, aldosterone might play a role in volume derangement and hypertension, even in early CKD. One might wonder whether there is a further rise in aldosterone as renal function declines, and if so, whether this translates into hypertension. Future research is imperative to investigate the link between creatinine clearance and high aldosterone across all spectra of renal function impairment, and what would be the implications for blood pressure treatment, in particular the role of MRA treatment on top of RAASi.

The primary limitation to our study was the post-hoc design. However, the robustness of our findings is supported by the independent study in which we found that the correlation between renal function and PAC was similarly present during RAASi with ACEi, and during dual RAASi. Furthermore, in our study the addition of MRA was not investigated.

## Conclusions

Our data show a consistent association between worse renal function and higher plasma aldosterone concentration, related to a higher ARR, during RAASi with either ARB, ACEi or both. Consequently, our data support the involvement of high aldosterone levels in hypertension in CKD with mildly impaired renal function – and show that a treatment regimen of RAASi combined with stepwise sodium intervention might be effective in correcting blood pressure.

## Additional files

Additional file 1: Table S1. Patient characteristics. This Table depicts the patients characteristics of patients included in the study. (DOCX 19 kb)

Additional file 2: Table S2. Multivariate analysis on the determinants of the plasma aldosterone concentration during placebo and during losartan treatment. In this table we illustrate the association between creatinine clearance and the plasma aldosterone concentration during placebo and losartan treatment, while adjusting for age and gender. (DOCX 13 kb)

## Abbreviations

ACEi: Angiotensin converting enzyme inhibitor
APRC: Active plasma renin concentration
ARB: Angiotensin II receptor blocker
ARR: Aldosterone-to-renin ratio
CKD: Chronic kidney disease
CrCl: Creatinine clearance
FGF23: Fibroblast growth factor 23
HCT: Hydrochlorothiazide
LN: Natural logarithm
LS: Low dietary sodium intake
MRA: Mineralocorticoid receptor antagonist
PAC: Plasma aldosterone concentration
RAASi: Renin-angiotensin-aldosterone system inhibition
RS: Regular dietary sodium intake
SBP: Systolic blood pressure
