# Supplementary material for: Renoprotective RAAS inhibition does not affect the association between worse renal function and higher plasma aldosterone levels
Source: BMC Nephrol. 2017 Dec 20;18:370. doi: 10.1186/s12882-017-0789-x (PMC5738866; doi:10.1186/s12882-017-0789-x)
Supplement: Supplementary file 1 — Patient characteristics. This Table depicts the patients characteristics of patients included in the study. (DOCX 19 kb) [file 12882_2017_789_MOESM1_ESM.docx]

| **Table S1 Patient characteristics** | | |  |
| --- | --- | --- | --- |
|  |  | **Current study (n=33)** | **Independent confirmation study (n=45)** |
| Mean age (years) | | 50 ± 12 | 51 ± 14 |
| Male (%) | | 24 (73) | 38 (84) |
| Mean BMI (kg/m²) | | 27.5 ± 4.4 | 27.4 ± 4.1 |
| Renal diagnosis | |  |  |
|  | IgA nephropathy (%) | 5 (15) | 12 (27) |
|  | Focal segmental glomerulosclerosis (%) | 7 (21) | 13 (29) |
|  | Membranous nephropathy (%) | 7 (21) | 8 (18) |
|  | Hypertensive nephropathy (%) | 5 (15) | 6 (13) |
|  | Other/inconclusive (%) | 9 (27) | 6 (13) |
| Mean urinary sodium excretion (mmol/24 hours) | | 200 ± 57 | 173 ± 73 |
| eGFR (ml/min) | | 60 ± 20 | 60 ± 27 |
| Creatinine clearance (ml/min) | | 85 (75-95) | 68 (58-79) |
| Proteinuria (g/day) | | 3.2 (2.5-4.0) | 1.6 (1.2-2.1) |
