# Supplementary material for: Renoprotective RAAS inhibition does not affect the association between worse renal function and higher plasma aldosterone levels
Source: BMC Nephrol. 2017 Dec 20;18:370. doi: 10.1186/s12882-017-0789-x (PMC5738866; doi:10.1186/s12882-017-0789-x)
Supplement: Supplementary file 2 — Multivariate analysis on the determinants of the plasma aldosterone concentration during placebo and during losartan treatment. In this table we illustrate the association between creatinine clearance and the plasma aldosterone concentration during placebo and losartan treatment, while adjusting for age and gender. (DOCX 13 kb) [file 12882_2017_789_MOESM2_ESM.docx]

**Table S2 Multivariate analysis on the determinants of the plasma aldosterone concentration during placebo and during losartan treatment**

| **PLACEBO** |  |  |  |  |
| --- | --- | --- | --- | --- |
|  | Model 1 | | Model 2 | |
|  | β | P-value | β | P-value |
| LN Creatinine clearance (ml/min) | -1.213 | 0.008 | -1.221 | 0.013 |
| Age (years) |  |  | 0.003 | 0.795 |
| Gender (Women) |  |  | -0.149 | 0.618 |
|  | *R*² | *0.211* |  | *0.220* |
|  | *Sig* | *0.008* |  | *0.069* |
|  |  |  |  |  |
| **LOSARTAN** | Model 1 | | Model 2 | |
|  | β | P-value | β | P-value |
| LN Creatinine clearance (ml/min) | -1.090 | 0.010 | -0.973 | 0.041 |
| Age (years) |  |  | 0.004 | 0.777 |
| Gender (Women) |  |  | 0.216 | 0.572 |
|  | *R*² | *0.203* |  | *0.212* |
|  | *Sig* | *0.010* |  | *0.079* |
